# Relationships between emotional intelligence, mental resilience, and adjustment disorder in novice nurses: a cross-sectional study in China

**DOI:** 10.3389/fpubh.2025.1567252

**Published:** 2025-06-13

**Authors:** Man Peng, Meijuan Xu, Hui Yang, Qiuxuan Zhang, Lijun Lai, Yanmei Liu, Qimei Xie, Xuexia Ma, Xiaoqun Mao

**Affiliations:** ^1^Department of Urology, Sun Yat-sen Memorial Hospital, Sun Yat-sen University, Guangzhou, China; ^2^Department of Nursing, Sun Yat-sen Memorial Hospital, Sun Yat-sen University, Guangzhou, China

**Keywords:** new nurses, emotional intelligence, mental resilience, adjustment disorder, mental health

## Abstract

**Objective:**

This study aims to explore the relationship among adjustment disorder, emotional intelligence, and mental resilience in newly licensed registered nurses.

**Method:**

This study adopts a cross-sectional design to explore the factors influencing work adaptation and mental resilience among newly graduated nurses with <3 years of clinical experience. The research was conducted by distributing a comprehensive, multi-part questionnaire to a targeted sample of new nurses across various healthcare settings. The questionnaire was meticulously designed to capture a holistic view of the participants' personal, professional, and psychological profiles, which included the Personal and Professional Characteristics Questionnaire, the Work Adaptation Difficulties Scale, the Mental Resilience Scale, and the 10-item Connor-Davidson Resilience Scale.

**Results:**

A total of 445 new nurses completed the questionnaire. The mean age of participants was 24.50 ± 2.77 years. Adjustment disorder negatively affected mental resilience (*r* = −0.460^**^, *P* < 0.001) and emotional intelligence (EI) (*r* = −0.380^**^, *P* < 0.001). Conversely, mental resilience positively influenced emotional intelligence (*r* = 0.714^**^, *P* < 0.001). The emotional perception (EP) dimension was negatively correlated with adjustment disorder (*r* = −0.396^**^, *P* < 0.001) but positively associated with increased mental resilience (*r* = 0.702^**^, *P* < 0.001).

**Conclusion:**

Findings suggest that higher levels of emotional intelligence and psychological resilience contribute significantly to enhanced work adaptation and the mitigation of psychological stress among nurses. Furthermore, job satisfaction appears to be a key predictor in the onset of adjustment disorders. These results underscore the necessity for head nurses and hospital administrators to adopt proactive measures aimed at promoting the psychological well-being of nursing professionals. Targeted interventions that foster emotional resilience and job satisfaction may serve as effective strategies to prevent maladaptive adjustment outcomes in high-stress clinical environments.

## 1 Introduction

Newly employed nurses (NEN) often encounter various challenges when entering clinical practice, including work pressure, time management, insufficient application of clinical skills, and communication issues with colleagues and patients (1, 2). These challenges can result in difficulties with adaptation, ultimately impacting nurses' job performance and mental health (3). Adaptation difficulties not only contribute to anxiety and depression but may also lead to high turnover rates, which can adversely affect the quality of nursing care (4). An increasing number of new nurses are entering the global labor force upon graduating from college; however, a significant proportion leave the profession due to workload stress, job dissatisfaction, and burnout (5). More than 50% of nurses report experiencing negative physical and emotional issues, often attributed to medical errors and their heavy workload (6). Situation like that is especially true for newly licensed registered nurses (NLRNs), who experience higher levels of stress and stress-related illnesses (7). Up to 66% of NLRNs report experiencing severe levels of burnout in their first years of practice (8). Research findings indicate that a substantial proportion of newly qualified nurses experience elevated levels of burnout and depression, leading to adverse health outcomes during their initial year of professional practice (9, 10). A Cox regression model indicated that a higher incidence of insomnia among nurses is associated with shorter employment duration (11). The primary concern among depressed newly licensed registered nurses is their inability to deliver patient care effectively and efficiently (9). Nurses with depression struggle to perform mental tasks and manage interpersonal responsibilities, are more prone to accidents, and exhibit reduced work output (12). These assertions not only highlight the personal struggles faced by depressed newly licensed registered nurses but also emphasize the detrimental effects on the quality of patient care (9). Studies have indicated that newly graduated nurses often possess inadequate professional knowledge and skills to effectively navigate diverse situations, including proficient communication with both patients and colleagues (13, 14). New nurses who worked in 3–5 months have more intentions leaving their jobs (5). Studies showed that the level of income, work environment, and job satisfaction are influencing factors in nurses' mental health (like depress, anxiety, and emotional intelligence) (15, 16).

The term emotional intelligence (EI) was initially introduced by Salovey and Mayer in 1990 to delineate a form of intelligence encompassing capacities to comprehend and manage one's own emotions, as well as those of others, and to leverage this comprehension to steer one's cognitive processes and behaviors (17). Higher EI is linked to greater psychological adjustment, resilience, and job satisfaction (18–20). On the contrary, lower EI is associated to great burnout and stress (21, 22). EI is also conformed a strong positive factor for mental health across population in a meta-analysis (23). EI has been extensively studied in nursing education. A literature review highlighted that EI confers multiple advantages for nursing students, with the potential to positively influence future applications within nursing education (24). A study of 1,744 Chinese nurses concluded that higher EI positively enhances nurses' work engagement (25).

Mental resilience is regarded as a dynamic progress, which would help the individual adapt to serious adversities, maintain mental health or recover from challenges actively (26). A previous study reported that 40.58% of nurses experienced mental disorders such as insomnia, anxiety, and depression (27). And a study in Chinese nurse students detected that positive mental resilience was associated with professional identity in Chinese nursing students on clinical placement, mediated through resilience and nurse-patient relationship. What's more, it was also a potential mechanism for nurse professional development (28). The nursing profession is inherently high-pressure, especially for newly graduated nurses as they transition into clinical practice. This stress is attributed to heavy workloads, emotional demands, shift rotations, and sudden role adjustments—factors that increase susceptibility to adjustment disorder (AD) (29, 30). Adjustment disorder among new nurses may lead to increased turnover intention, higher rates of medical errors, and even mental health crises such as burnout in China.

The aim of this study is to explore the relationship among adjustment disorder, emotional intelligence, and mental resilience in newly licensed registered nurses. And the primary hypothesis is that there would be a significant positive relationship between emotional intelligence and mental resilience, and a negative relationship with adjustment disorder among novice nurses in China. Besides, emotional intelligence may positively influence mental resilience, which in turn negatively affects adjustment disorder among novice nurses.

## 2 Method

### 2.1 Study design

This is a descriptive and cross-sectional study.

### 2.2 Participants and research tools

#### 2.2.1 Participants

This study was conducted in several hospitals in Guangdong Province, China. A total of 445 new nurses, each with no more than 3 years of working experience, were selected to complete the questionnaires. Data were collected from August to October 2024. On the Wenjuanxing platform, the questionnaire content was sequentially entered and verified for accuracy before creating a code to collect data. Prior to distributing the questionnaire, the researcher obtained consent from the hospital head nurses, explained the purpose of the survey, and secured informed consent from the participants. The questionnaire was completed anonymously, and participants voluntarily fill it out online via WeChat.

The questionnaire used standardized instructions to inform participants of the research purpose, filling methods, and precautions. Participants were required to answer truthfully based on their actual situations. The questionnaires were designed to allow for resuming from where they left off, enabling participants to automatically continue from the last unanswered section when they re-enter the questionnaire. To prevent duplicate responses, each IP address was restricted to one submission. After collecting the questionnaires, a time control has been implemented, with each questionnaire required to be completed within 10 min. Questionnaires that do not meet the requirements have been filtered out to ensure the authenticity and reliability of the sample data.

The scale consists of 21 dimensions, multiplied by 20 times as 21 × 20 = 420. Accounting for a 10% questionnaire failure rate, 420 × 0.1 = 42, yielding a projected total of 420 + 42 = 462. However, during data cleaning in the analysis process: 17 invalid questionnaires were excluded (3.68% of total); Final analytical sample: valid responses *N* = 445. After the review was completed, the data were downloaded and imported into SPSS (version: 27.0) for analysis. This research was approved by the ethics committee of Sun Yat-sun Memorable Hospital, Sun Yat-sun University (Approved No. SYSKY-2024-1113-01). Every participant in this research had been told the aim of the study.

Inclusion criteria of participants.

1. Possession of a nurse nursing license.
2. Engagement in clinical work for 1–36 months.
3. Age between 20 and 30 years.

Exclusion criteria of participants.

1. Nurses from other hospitals undergoing advanced training.
2. Incomplete questionnaire responses.
3. Nurses on leave or not on duty, including those on sick leave, marriage leave, maternity leave, or out for further education.

#### 2.2.2 Research tools

Four questionnaires were designed to collect data. The section on personal and professional characteristics includes questions about age, gender, duration of professional experience, unit, and whether they chose their profession and units voluntarily.

#### 2.2.3 Work adaptation difficulties scale

The Work Adaptation Difficulties Scale (Work Attitude Scale, Wa), developed by Tyollaska in 1942, was used to measure the work adaptation of nursing interns. This scale reflects an individual's perspectives and interests regarding their reality and intrinsic motivations. It consists of a single dimension with 37 items: 29 are scored positively (1 point for “Yes”) and 8 are scored negatively (1 point for “No”). The total score ranges from 0 to 37, with higher scores indicating a greater lack of work motivation and more significant work adaptation difficulties. Research by scholars such as Ji Shumao has demonstrated that this scale possesses good reliability and validity, with a Cronbach's alpha coefficient of 0.77.

#### 2.2.4 10-item Connor–Davidson resilience scale (CD-RISC-10)

The Mental Resilience Scale was developed by American psychologists Connor and Davidson in 2003 (31). The original version of 10-item Connor-Davidson Resilience Scale (CD-RISC-10) was coined by Campbell-Sills and Stein (32) and translated into Chinese version in 2010 (27), which contains 10 items assessed using a 5-point Likert scale, ranging from 0 to 4, which correspond to “Never, Rarely, Sometimes, Often, Almost Always.” Each item is scored from 0 to 4, with total scores ranging from 0 to 40. Scores below 10 indicate low resilience, scores between 10 and 30 indicate moderate resilience, and scores above 30 indicate high resilience. Higher scores reflect better psychological resilience. The reliability coefficient of the scale was verified to be 0.879, indicating a high level of reliability and validity.

#### 2.2.5 Emotional intelligence scale

The Emotional Intelligence Scale (EIS) was developed by Schutle et al. based on Mayer and Salovey's theory of emotional intelligence. The Chinese version of the EIS was translated by Wang Caikang in 2002 and consists of 33 items. It includes four dimensions: Emotional Perception (items 2, 3, 9, 12, 13, 14, 16, 22, 23, 28, 30, 31), Emotional Expression (items 1, 5, 15, 25, 33), Self-Emotional Management (items 7, 8, 10, 17, 20, 24), and Other's Emotional Management (items 4, 6, 11, 18, 19, 21, 26, 27, 29, 32), with items 5, 28, and 33 being reverse scored. The scale employs a 5-point Likert scoring system, where scores range from 1 to 5, corresponding to “Strongly Disagree” to “Strongly Agree.” Higher scores indicate higher emotional intelligence. Preliminary studies have shown that the scale has a Cronbach's alpha coefficient of 0.87, indicating good reliability and validity for this study.

#### 2.2.6 Data analysis

Statistical Package for the Social Sciences (SPSS) version 27 was used to analyze the data. Data analysis was performed using mean, standard deviation, frequency, percentage, chi-square, *t*-test, bivariate correlation analysis, one-way analysis of variance (ANOVA) and the Schefft test.

Descriptive analysis: this was employed to describe the basic characteristics of clinical nurses, including demographic information, emotional intelligence, work adaptation, and psychological resilience, as measured by various scales. Continuous data were presented as mean ± standard deviation (X ± s), while categorical data were described using frequency and composition ratio (%).

Univariate analysis: this method was used to compare differences in work adaptation scores among clinical nurses with different demographic characteristics. For normally distributed data that met homogeneity of variance, *t*-tests and analysis of variance (ANOVA) were applied. When the data did not conform to normal distribution or homogeneity of variance, rank-sum tests were utilized.

Correlation analysis: pearson correlation analysis was employed to analyze the relationships among emotional intelligence, work adaptation, and psychological resilience in new nurses.

## 3 Results

Table 1 shows the demographic information. The mean age of the new nurses in this study was 24.50 ± 2.77 years old; 400 (89.3%) of all participants were female. 401 (90.1%) of them were unmarried and 44 (9.9%) were married. Furthermore, a total of 138 (31.0%) participants reported monthly incomes below ¥5,000, 288 (64.7%) earned between ¥5,000–10,000, and 19 (4.3%) had incomes exceeding ¥10,000. Among all participants, 4 (0.9%) held vocational nursing diplomas (secondary vocational education), 121 (27.2%) graduated with associate degrees in nursing, 312 (70.1%) held bachelor's degrees in nursing (BSN), and 8 (1.8%) had obtained master's degrees. Lastly, 109 (24.5%) worked in the internal medicine, 236 (77.5%) worked in the surgical department, 16 (3.6%) worked in the genecology department and 20 (4.5%) worked in pediatric services. 10 (2.2%) of new nurses worked in emergency unit and 19 (4.2%) worked in the intensive care unit (ICU), and 8 (1.8%) worked in operating room. 2 (0.4%) worked in outpatient department while the other 25 (5.6%) worked in other departments like Chinese medicine departments, dentistry department or ophthalmology.

**Table 1:** Demographic information.

**Items**	**Mean (SD)/number**	**Percentage**
**Year**	24.50 ± 2.77	
**Marital status**		
Married	44	9.9%
Unmarried	401	90.1%
**Sex**		
Male	45	10.1%
Female	400	89.9%
**Income/month**		
< 5000	138 (26.65 ± 7.5)	31.0%
5000–10000	288 (25.63 ± 7.0)	64.7%
>10000	19 (30.26 ± 6.8)	4.3%
**Educational level**		
Vocational diploma nurse	4	0.9%
Associate degree nurse	121	27.2%
Bachelor's degree nurse	312	70.1%
Graduate degree	8	1.8%
**Single child**		
Yes	66	14.8%
No	379	85.2%
**Night shift/month**		
0	32	7.2%
1–5	172	38.7%
6–10	205	46.1%
>10	36	8.1%
**Working ward**		
Internal medicine	109	24.5%
Surgical department	236	77.5%
Genecology department	16	3.6%
Pediatric services	20	4.5%
Emergency unit	10	2.2%
ICU	19	4.2%
Operating room	8	1.8%
Outpatient department	2	0.4%
Other	25	5.6%

Table 2 presents psychometric scores across six key domains. Data of participants reported the median score of adjustment disorder is 9 (possible range: 0–34), indicating relatively low symptom severity in this sample. Median score of mental resilience is 27 (range: 0–40), suggesting moderate-to-high resilience levels. And there is a high average score of total emotional intelligence (124.17 ± 19.65, range: 45–160). Additionally, the mean scores for the four dimensions of emotional intelligence were as follows: Emotional Perception (EP): (45.24 ± 6.92, range: 16–60). Emotional Expression (EE): Narrow score distribution (16.65 ± 1.77, range: 11–24), suggesting limited variability. Self-Emotion Management (SEM): (23.38 ± 3.88, range: 6–30). And others' Emotion Management (OEM): Highest mean among subscales (37.19 ± 6.16, range: 10–50).

**Table 2:** Score of variables.

**Item**	**Mean ±SD**	**Median**	**Score range**
AD		9	0–34
MR		27	0–40
EI	124.17 ± 19.65		45–160
EP	45.24 ± 6.92		16–60
EE	16.65 ± 1.77		11–24
SEM	23.38 ± 3.88		6–30
OEM	37.19 ± 6.16		10–50

AD, adjustment disorder; MR, mental resilience; EI, emotional intelligence; EP, emotional perception; EE, emotional expression; SEM, self-emotional management; OEM, other's emotional management.

Table 3 shows the relationship between different variables. Spearman correlation analysis revealed that adjustment disorder plays a negative influence in mental resilience (*r* = −0.460^**^, *P* < 0.001), and adjustment disorder also plays a negative role in influencing emotional intelligence (EI) (*r* = −0.380^**^, *P* < 0.001). This result indicates that individuals facing adjustment disorders may experience higher psychological stress, thereby reducing their mental resilience. Emotional intelligence involves the ability to perceive, understand, and manage emotions, and adjustment disorders may lead to a decline in an individual's emotion regulation capacity. Besides, mental resilience plays a positive role in influencing emotional intelligence (*r* = 0.714^**^, *P* < 0.001). This suggests that individuals with higher mental resilience tend to also possess higher emotional intelligence. Since the Emotional Intelligence Scale has four dimensions, the scores of these four dimensions were compared with the scores of adaptation disorder and mental resilience in this study. Firstly, dimension emotional perception (EP) and adjustment disorder are negatively correlated (*P* < 0.001, *r* = −0.396^**^), but a positive factor in increasing mental resilience (*r* = 0.702^**^, *P* < 0.001). Secondly, dimension emotional expression (EE) shows a bad factor in increasing the level of adjustment disorder (*r* = −0.220^**^, *P* < 0.001) while a good influence for improving mental resilience (*r* = 0.375^**^, *P* < 0.001). And there is a negative correction between dimension self-emotional management (SEM) and adjustment disorder (*r* = −0.321^**^, *P* < 0.001) and a positive relationship exists between SEM and mental resilience (*r* = 0.631^**^, *P* < 0.001). Lastly, dimension other's emotional management (OEM) shows a negative correction with adjustment disorder (*r* = −0.328^**^, *P* < 0.001) and a positive correction with mental resilience (*r* = 0.687^**^, *P* < 0.001). Individuals who can effectively manage their own emotions are less likely to experience adjustment disorders and more likely to have higher mental resilience.

**Table 3:** Influence factor between different variables.

**Item**	**AD**	**MR**	**EI**	**EP**	**EE**	**SEM**	**OEM**
AD	1	−0.460^**^	−0.380^**^	−0.396^**^	−0.220^**^	−0.321^**^	−0.328^**^
MR	−0.460^**^	1	0.714^**^	0.702^**^	0.375^**^	0.631^**^	0.687^**^

^*^P < 0.05, ^**^P < 0.001.

AD, Adjustment disorder; MR, Mental resilience; EI, Emotional intelligence; EP, Emotional perception; EE, Emotional expression; SEM, Self-emotional management; OEM, Other's emotional management.

Table 4 showed the results of multiple regression analysis in adaptation disorder. The goodness of fit *R*^2^ = 0.288, Adjusted *R*^2^ = 0.281, indicating that the predictors (resilience, emotional intelligence, job satisfaction, and work environment) collectively explain 28.8% of the variance in work adaptation scores. The model is statistically significant (implied by *R*^2^ and predictor *P*-values). Durbin-Watson statistic ≈1.94 (close to 2) suggests no significant autocorrelation in residuals.

**Table 4:** Multiple regression analysis (Dependent variable: adaptation disorder scores).

**Variable**	***B* (SE)**	**β**	***t***	***P***	**95% CI**	**VIF**
Constant	27.54 (1.98)	–	13.88	< 0.001	[23.63, 31.45]	–
Job satisfaction	−1.97 (0.44)	−0.224	−4.43	< 0.001	[−2.84, −1.10]	1.57
Work environment	−0.85 (0.56)	−0.074	−0.074	−1.50	[−1.95, 0.25]	1.50
Mental Resilience	−0.27 (0.05)	−0.303	−5.43	< 0.001	[−0.37, −0.17]	1.92
Emotional intelligence	−0.03 (0.02)	−0.078	−1.41	0.160	[−0.07, 0.01]	1.89

*R*^2^ = 0.288, Adjusted *R*^2^ = 0.281, *P* < 0.001.

The significant predictors showed in Table 4 are as follow: Job satisfaction (β = −0.224, *P* < 0.001), each 1-unit increase in satisfaction reduces adaptation scores by 1.97. Resilience (β = −0.303, *P* < 0.001) means higher resilience predicts lower adaptation. However, work environment and emotional intelligence are non-significant predictors (*P* > 0.05). And all VIFs < 2, which indicating no concerning collinearity. Adaptation disorder correlated negatively with resilience (*r* = −0.467), job satisfaction (*r* = −0.426), and work environment (*r* = −0.325). Emotional intelligence subscales (e.g., emotion perception, management) showed high correlations with resilience (*r* > 0.6), potentially masking significance in regression due to multicollinearity.

The multiple regression analysis revealed that mental resilience and job satisfaction were significant predictors of lower work adaptation disorder scores (β = −0.303 and −0.224, respectively; *P* < 0.001), whereas work environment and emotional intelligence had no significant effects. The model accounted for 28.1% of the variance (Adjusted *R*^2^ = 0.281) with no multicollinearity concerns (all VIFs < 2). Interventions targeting resilience and job satisfaction may mitigate adaptation among new nurses.

The analysis also revealed a significant effect of income level on psychological resilience (*F* = 4.37, *P* = 0.013). *Post hoc* comparisons using Tukey's HSD test demonstrated participants with Income Level 2 showed significantly lower resilience scores compared to Income Level 3 (M_diff_ = −3.21, 95% CI (−5.89, −0.53), *P* = 0.017). No other pairwise comparisons reached statistical significance (all *P* > 0.05).

## 4 Discussion

This study aims to explore the interrelationship among adjusting disorder, emotional intelligence, and mental resilience in newly employed nurses. By analyzing the interactions among these factors, valuable insights can be provided for nursing management and education.

It is noteworthy that when the level of emotional intelligence is high, the prevalence of mental resilience is significantly greater among new nurses. That means nurses with high emotional intelligence are better able to understand and manage their own emotions, which can reduce anxiety and unease caused by work-related stress, thereby lowering the risk of adaptation difficulties. Therefore, nursing education institutions and hospital managing departments should prioritize developing the EI of new nurses through relevant training and courses to help them enhance their emotional management skills.

In this study, the sub-dimensions of emotional intelligence—emotional perception, self-emotional management, and others' emotional management—were found to significantly enhance mental resilience (*r* > 0.6, *P* < 0.001). This indicates that new nurses should focus on improving their abilities in these areas of emotional intelligence. Additionally, hospital management and nursing education institutions should pay more attention to these aspects to support the development of emotional intelligence among new nurses. The total emotional intelligence score and the scores of its sub-dimensions all positively contribute to reducing adjustment disorders among new nurses in this study. Nurses with high EI are better at building supportive relationships, especially those with higher dimension other's emotional management, they tend to have higher mental resilience in this study. This is similar to another research in Jordan, which indicated that higher emotional intelligence is associated with higher mental resilience (16).

The findings indicate that newly graduated nurses exhibited a reduction in adjustment disorders when possessing elevated levels of emotional intelligence. This implies that individuals within the nursing profession who demonstrate heightened emotional intelligence have a beneficial influence on their capacity to acclimate to novel occupational settings. This conclusion is similar to a meta-analysis that examined the relationship between emotional intelligence and optimism across 6,889 participants from 25 independent samples, finding that a higher level of emotional intelligence was associated with greater optimism (23). Additionally, it has been verified that emotional intelligence is linked to personal disorders in college students. Negative emotions or experiences, such as anxiety, fearfulness, dramatic behaviors, and erratic moods, can influence emotional intelligence (33). Prior research showed that the overall and core mental health level of Chinese nurses were at a moderate level (34).

The results of this study also indicate that job satisfaction among new nurses can affect their adaptation disorders, with higher job satisfaction reducing adaptation difficulties. Systematic reviews show that higher job satisfaction in new nurses can enhance nurses' mental health, reduce negative emotions, and improve the quality of nursing and patient care (35). *Post hoc* analysis with Tukey's HSD revealed significantly lower psychological resilience in income level 2 (5,000–10,000/month) compared to level 3 (>10,000/month) but unexpectedly found no differences between level 1 (< 5,000/month) vs. 2/3, suggesting a threshold effect at mid-tier income. The reason for these results may be regional disparities in data collection. An income of ¥5,000–10,000 may only cover basic living costs in some high-expense cities (e.g., Guangzhou/Shenzhen), while in less developed cities with lower prices (e.g., Foshan/Zhongshan), even an income < ¥5,000 can meet the daily expenses of new nurses due to lower prices. Future studies should collect stratified data from cities with different economic levels to analyze the impact of income on psychological resilience.

Multiple regression analysis in this study showed that resilience (β =-0.303, *P* < 0.001) and job satisfaction (β = −0.224, *P* < 0.001) were established as the core protective factors for job adjustment disorder in new nurses, which jointly explained 28.1% of the variation. This is consistent with prior studies, which concluded that job satisfaction was negatively related to nurses' turnover intentions and positively correlate with good quality of nursing care (36–38).

This study also identified that mental resilience negatively impacts adjustment disorders; as mental resilience improves, adjustment disorders decrease. This indicates that new nurses should focus on enhancing their mental resilience to reduce adjustment disorders. In the future, hospital management should pay attention to the cultivation of psychological resilience of new nurses and establish a highly satisfactory working atmosphere in the department.

## 5 Conclusion

In summary, higher levels of emotional intelligence and psychological resilience are associated with improved work adaptation and reduced psychological stress among nurses. Job satisfaction has also been identified as a critical factor influencing the development of adjustment disorders. These findings highlight the importance of prioritizing mental health within clinical settings. It is essential that head nurses and hospital administrators implement supportive strategies aimed at strengthening nurses' psychological resilience and promoting job satisfaction, thereby contributing to the prevention of adjustment-related difficulties in the workplace.

## 6 Advantages and shortages

This research explored the relationships among adjustment disorders, mental resilience, and emotional intelligence, marking the first study to examine how these three factors interact in new nurses. Adjustment disorders can significantly hinder new nurses in coping with their roles during the first 3 years of practice. This study identifies key factors that influence job adjustment for new nurses, providing a foundation for future interventional studies.

However, it is important to note that this research was conducted in only one province in China with a limited sample size of 445 participants. Future studies should aim to include a broader range of hospitals across multiple provinces and involve more participants. Additionally, this study utilized only three questionnaires, limiting the identification of influential factors affecting new nurses. More comprehensive research tools are needed to uncover additional factors that may affect job adjustment for new nurses in the future. As a cross-sectional study, this research cannot establish causal relationships between emotional intelligence and adjustment disorders, nor capture dynamic changes in nurses' adaptation processes over time. Adopt longitudinal designs to establish causality in the future.

## Data Availability

The original contributions presented in the study are included in the article/supplementary material, further inquiries can be directed to the corresponding authors.
